# Healthcare professionals’ perspectives on implementation of universal tumor DNA testing in ovarian cancer patients: multidisciplinary focus groups

**DOI:** 10.1007/s10689-022-00294-0

**Published:** 2022-05-16

**Authors:** Vera M. Witjes, Jozé C. C. Braspenning, Nicoline Hoogerbrugge, Yvonne H. C. M. Smolders, Dorien M. A. Hermkens, Marian J. E. Mourits, Marjolijn J. L. Ligtenberg, Margreet G. E. M. Ausems, Joanne A. de Hullu

**Affiliations:** 1 Department of Human Genetics, Radboud Institute for Molecular Life Sciences, Radboud University Medical Center, Nijmegen, The Netherlands; 2 Scientific Center for Quality of Healthcare, Radboud Institute for Health Sciences, Radboud University Medical Center, Nijmegen, The Netherlands; 3 Department of Human Genetics, Radboud University Medical Center, Nijmegen, The Netherlands; 4 Department of Gynecology, University Medical Center Groningen, University of Groningen, Groningen, The Netherlands; 5 Department of Pathology, Radboud Institute for Molecular Life Sciences, Radboud University Medical Center, Nijmegen, The Netherlands; 6 Division Laboratories, Pharmacy and Biomedical Genetics, Department of Genetics, University Medical Center Utrecht, Utrecht, The Netherlands; 7 Department of Obstetrics and Gynecology, Radboud Institute for Health Sciences, Radboud University Medical Center, Nijmegen, The Netherlands

**Keywords:** Epithelial ovarian cancer, Heredity, *BRCA*, Integrated care, Tumor testing, Implementation

## Abstract

Universal tumor DNA testing in epithelial ovarian cancer patients can function not only as an efficient prescreen for hereditary cancer testing, but may also guide treatment choices. This innovation, introduced as Tumor-First workflow, offers great opportunities, but ensuring optimal multidisciplinary collaboration is a challenge. We investigated factors that were relevant and important for large-scale implementation. In three multidisciplinary online focus groups, healthcare professionals (gynecologic oncologists, pathologists, clinical geneticists, and clinical laboratory specialists) were interviewed on factors critical for the implementation of the Tumor-First workflow. Recordings were transcribed for analysis in Atlas.ti according to the framework of Flottorp that categorizes seven implementation domains. Healthcare professionals from all disciplines endorse implementation of the Tumor-First workflow, but more detailed standardization and advice regarding the logistics of the workflow were needed. Healthcare professionals explored ways to stay informed about the different phases of the workflow and the results. They emphasized the importance of including all epithelial ovarian cancer patients in the workflow and monitoring this inclusion. Overall, healthcare professionals would appreciate supporting material for the implementation of the Tumor-First workflow in the daily work routine. Focus group discussions have revealed factors for developing a tailored implementation strategy for the Tumor-First workflow in order to optimize care for epithelial ovarian cancer patients. Future innovations affecting multidisciplinary oncology teams including clinical geneticists can benefit from the lessons learned.

## Introduction

Due to rapid technological advancements over the years, genetic testing for hereditary cancer syndromes now plays an increasingly important role as a diagnostic tool in healthcare. The identification of individuals with a hereditary cancer syndrome enables cancer prevention or early intervention in patients and relatives. Furthermore, developments in cancer genetics are still ongoing and advances in molecular diagnostics may also offer great opportunities for personalized treatments [1, 2]. Innovations are not solely technical but they may also confer a change in the diagnostic workflow. These new innovations are challenging to implement in daily practice, as they involve tumor-specific changes in the collaboration among multidisciplinary teams of healthcare professionals (sometimes across different hospitals) involved in the diagnostics and treatment of a specific cancer, including clinical geneticists.

The Tumor-First approach, universal tumor DNA testing for all newly diagnosed epithelial ovarian cancer (OC) patients, is an example of a recent innovation in oncogenetics. Around 10 to 15% of all OC patients have a hereditary predisposition to develop the disease [3–5], leading to the recommendation that all OC patients are eligible for genetic counseling and germline testing [4, 6, 7]. A tumor DNA test, identifying germline and acquired pathogenic variants in *BRCA1/2* and other OC risk genes, functions as an efficient prescreen to tailor genetic counseling to patients at higher risk of hereditary cancer. Simultaneously, the test identifies patients that may benefit most from treatment with a PARP inhibitor [8]. The main advantage of this workflow is that it facilitates the counseling of genetic risk stratified on the results of the tumor DNA test shortly after diagnosis and that it may reduce disparities in access to genetic testing [9].

The Tumor-First workflow was regionally pilot tested in the Netherlands, and its evaluation revealed feasibility and appreciation by patients and gynecologists [3]. Nationwide implementation of the Tumor-First workflow comes with some challenges, including a change in the interdisciplinary collaborations between healthcare professionals and hospitals. Interdisciplinary team work in healthcare has been enthusiastically advertised as being beneficial for (cancer) patients [10, 11]. Tumor boards have been established in which various disciplines meet to discuss the management of cancer patients [12, 13]. However, this interdisciplinary teamwork is complex, as it involves overlapping professional areas. New interdisciplinary innovations, such as the Tumor-First workflow, could affect these boundaries [14, 15].

It is extremely useful to know which factors influence the implementation of the Tumor-First workflow. In listing these factors, the classification scheme of Flottorp can be used to distinguish factors related to (1) innovation itself, (2) patients, (3) healthcare professionals, (4) professional interactions, (5) incentives and resources, (6) organizations, and (7) social, political and legal issues [16]. Based on this classification, we aimed to identify factors critical for interdisciplinary largescale (nationwide) implementation of the Tumor-First workflow from healthcare professionals’ perspectives in order to develop a sustainable implementation strategy later on—one that would potentially be useful for future interdisciplinary innovations in the field of genetics.

## Materials and methods

### Study design

In a qualitative study, semi-structured online focus group interviews were held with professionals involved in the Tumor-First workflow—including gynecologic oncologists, pathologists, clinical geneticists, and clinical laboratory geneticists and clinical scientists in molecular pathology. The latter two groups are non-medical doctors specialized in genetic testing and data interpretation and will be referred to as clinical laboratory specialists. A group setting was preferred to stimulate multidisciplinary discussions among professionals. A focus group setting allowed participants to talk and discuss—freely but with some guidance—the factors critical for the implementation of the Tumor-First workflow. This study was assessed by the Medical Ethical Committee (CMO) of region Arnhem-Nijmegen, who declared there was no need for ethical approval (number 2021-7290). We report this study in accordance with the COREQ checklist [17].

### Setting

**Fig. 1:**
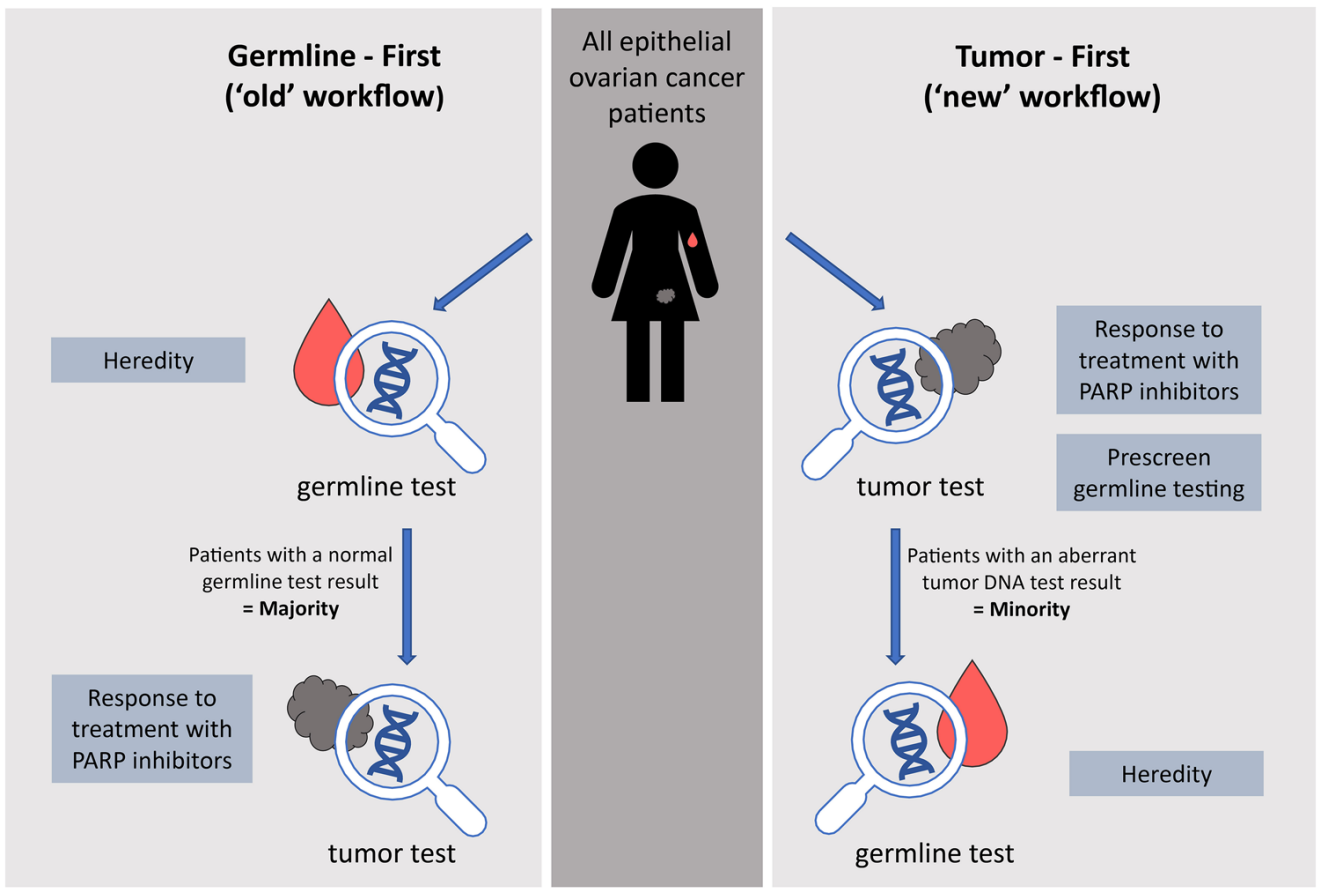
An overview comparing the ‘old’ workflow (germline-first) to the Tumor-First workflow. The Tumor-First workflow requires fewer patients to have both a tumor DNA test and a germline test (a majority was required in the old workflow [4, 5], compared to a minority in the Tumor-First workflow [3]). Implementation requires a shifting one’s orientation from the ‘old’ workflow to the Tumor-First workflow

In the Netherlands (population of 17 million), around 1300 patients are diagnosed yearly with OC [18]. Here, surgical care is centralized in gynecologic oncology centers and surgical procedures are performed by gynecologic oncologists (gynecologists who performed an additional 2 year subspecialty ‘gynecologic oncology’) [19]. There are nine gynecologic oncology centers (seven university hospitals, one non-academic hospital and one cancer-center), each having connections with regional hospitals. In previous national multidisciplinary meetings regarding genetic testing for OC, the Tumor-First workflow emerged as the preferred workflow to replace the ‘old’ one (all OC patients referred to the clinical geneticist for germline testing), as illustrated in Fig. 1. The level of experience with the Tumor-First workflow varies across the gynecologic oncology centers, varying from more than two years’ experience (three centers), one to two years’ experience (three centers), to less than a year of experience (three centers). The level of experience with the workflow and some general information were collected by telephoning gynecologic oncologists, pathologists and clinical geneticists from the expert centers.

### Recruitment of participants

We organized three focus groups between February 8th and March 22nd 2021 for all nine gynecologic oncology centers in the Netherlands. As the familiarity with the Tumor-First workflow varied somewhat between these centers, we strived for a mix of skills in each focus group. We invited a gynecologic oncologist, a clinical geneticist, a pathologist, and a clinical laboratory specialist (clinical laboratory geneticist or clinical scientist in molecular pathology) from every gynecologic oncology center. Representatives were selected in consultation with the centers. Additionally, all gynecologic oncologists and pathologists were asked to invite a colleague from a hospital in their region to participate. Invitations were sent per email. Professionals who were unable to attend were asked to delegate a colleague.

### Interview guide

**Fig. 2:**
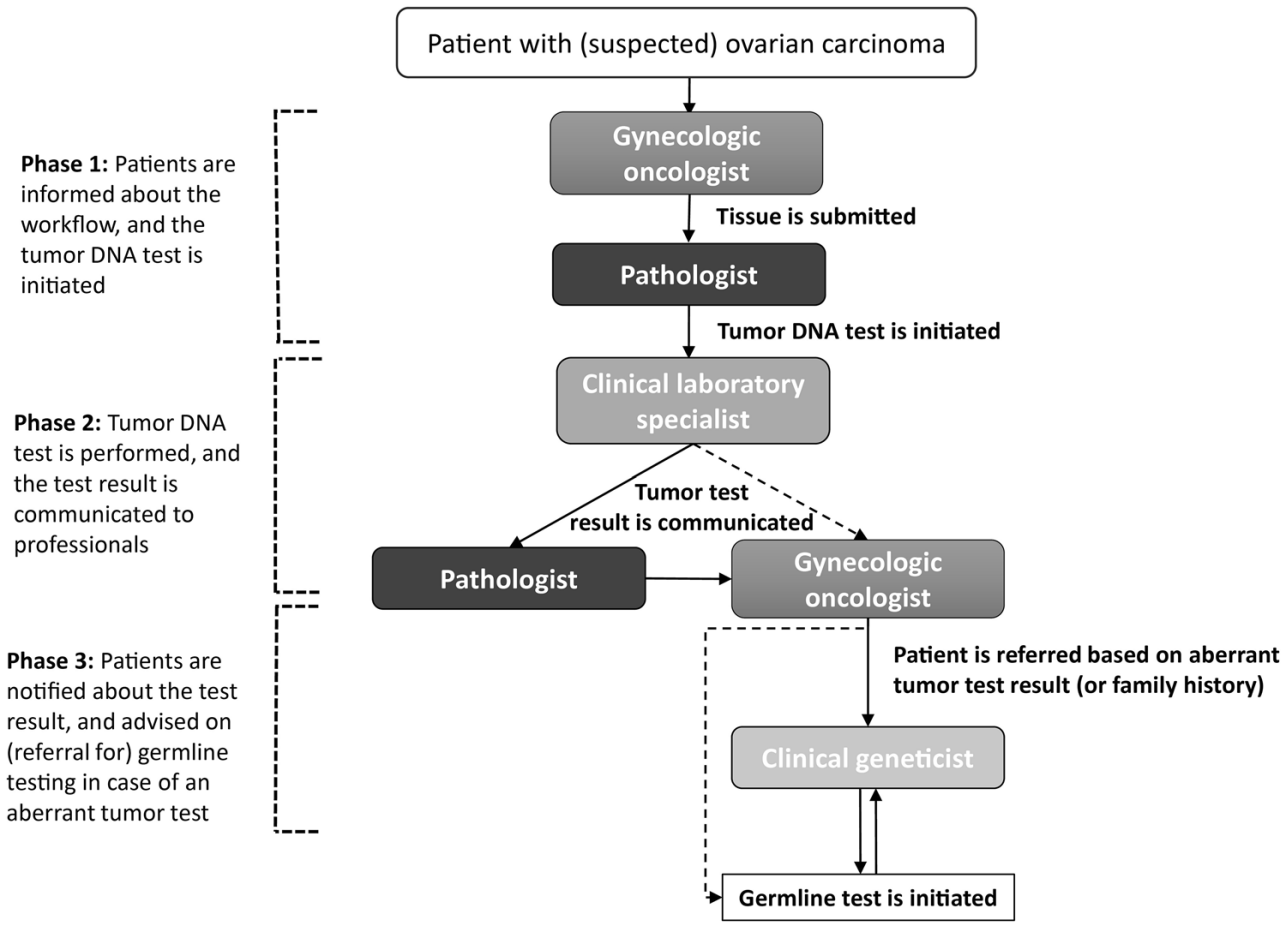
Tumor-First workflow divided into three phases used to discuss factors critical for implementation of the workflow during focus group discussions

A semi-structured interview guide was developed based upon the various phases of the Tumor-First workflow and the theoretical framework by Flottorp [16]. The interview guide distinguished the three phases of the workflow; (1) patients are informed about the workflow and the tumor DNA test is initiated, (2) tumor DNA test is performed and test result is communicated to professionals (3) patients are notified about the test result and advised on (a referral for) germline testing in case of an aberrant tumor test. We use the term ‘aberrant test’ in case a pathogenic variant in an OC risk gene is detected, and ‘normal test’ in case no pathogenic variant is detected. The phases included in the interview guide are illustrated in Fig. 2. For each phase we started to retrieve information about the current the workflow, followed by a discussion on possible barriers while exchanging solutions already considered. Possible barriers and enablers have been addressed in a free and open discussion by an experienced moderator (author: JB) who knew the seven domains from the framework of Flottorp by heart. In each focus group, two key persons in this field were present to support the moderator in answering specific questions from participants. Focus groups were conducted online using the Zoom videoconferencing platform, and lasted 90 to 120 min. The zoom meetings were recorded (video and audio) and transcribed non-verbatim. Participants were informed about the recording and were given the opportunity to object. All gave permission to be recorded. To ensure the privacy of participants, transcripts were deidentified before analysis. Original transcripts were in Dutch and quotes have been translated for this paper. Contextual additions to the quotes are displayed using […].

### Data analysis

The main results of the focus groups were summarized and sent to the participants (including those who were unable to attend) to allow them to provide comments and additions. Subsequently, the complete transcripts were uploaded to the program ATLAS.ti GmbH Version 8.4.20 to aid the qualitative analysis. Transcripts were analyzed deductively using a framework approach [20]. We prepared a framework combining the theory from Flottorp with the phases of the Tumor-First workflow [16]. After familiarization, reading and rereading of the first transcript, a first draft of codes was prepared by two project members independently (authors: VW, YS). Thereafter, the transcript was coded by both project members and, if necessary, codes were added, merged or altered. This process was repeated for the following two transcripts. Ultimately, a final codebook was prepared and transcripts were coded again by both project members to test reliability. Another author (JB) was consulted in case of disagreement. This codebook including relevant text passages was discussed to identify core themes regarding Tumor-First testing. These themes were connected to the seven implementation domains [16] for presentation in this report.

## Results

### Study population

Three focus groups were organized, and a total of 53 invitations to participate in one of those focus group discussions were sent out. Forty professionals accepted the invitation and participated, six declined, three did not reply and four dropped out after signing up. Table 1 provides an overview of the professional disciplines and number of years of experience with Tumor-First workflow of the participants in each focus group. In comparison, the participation of gynecologic oncologists was somewhat higher in the first and third focus group, whereas in the second focus group more pathologists participated. The level of experience was well distributed across the three focus groups.

**Table 1:** Profession and experience of healthcare professionals participating in three focus groups

	Focus group 1	Focus group 2	Focus group 3
	n = 13	n = 14	n = 13
Professional discipline			
Gynecologic oncologist	6	3	7
Pathologist	1	4	2
Clinical geneticist	3	3	2
Clinical laboratory specialist (pathology—genetics)	3 (1–2)	4 (3–1)	2 (2–0)
Experience Tumor-First			
> 2 years	5	5	3
1–2 years	4	4	5
0–1 year	4	5	5

### Tumor-First workflow: healthcare professionals’ endorsement

Healthcare professionals from all four disciplines (gynecologic oncologists, pathologists, clinical geneticists, and clinical laboratory specialists) have an optimistic view on the Tumor-First workflow for OC patients and they endorse national implementation. They were all familiar with the concept of the Tumor-First workflow and no questions were raised on the usefulness or feasibility of this diagnostic workflow. Healthcare professionals indicated that the main advantage is that a tumor DNA test provides information for both heredity and treatment. At the outpatient clinics, OC patients often have questions with regards to cancer risk for family members and with the Tumor-First workflow it can be reassuring that there is a standard pretest.

*Testing of tumors is very useful and provides a lot of information* **Clinical geneticist_6**

*It is interesting that it brings together consequences for treatment and for family* **Gynecologic oncologist_9**

**Fig. 3:**
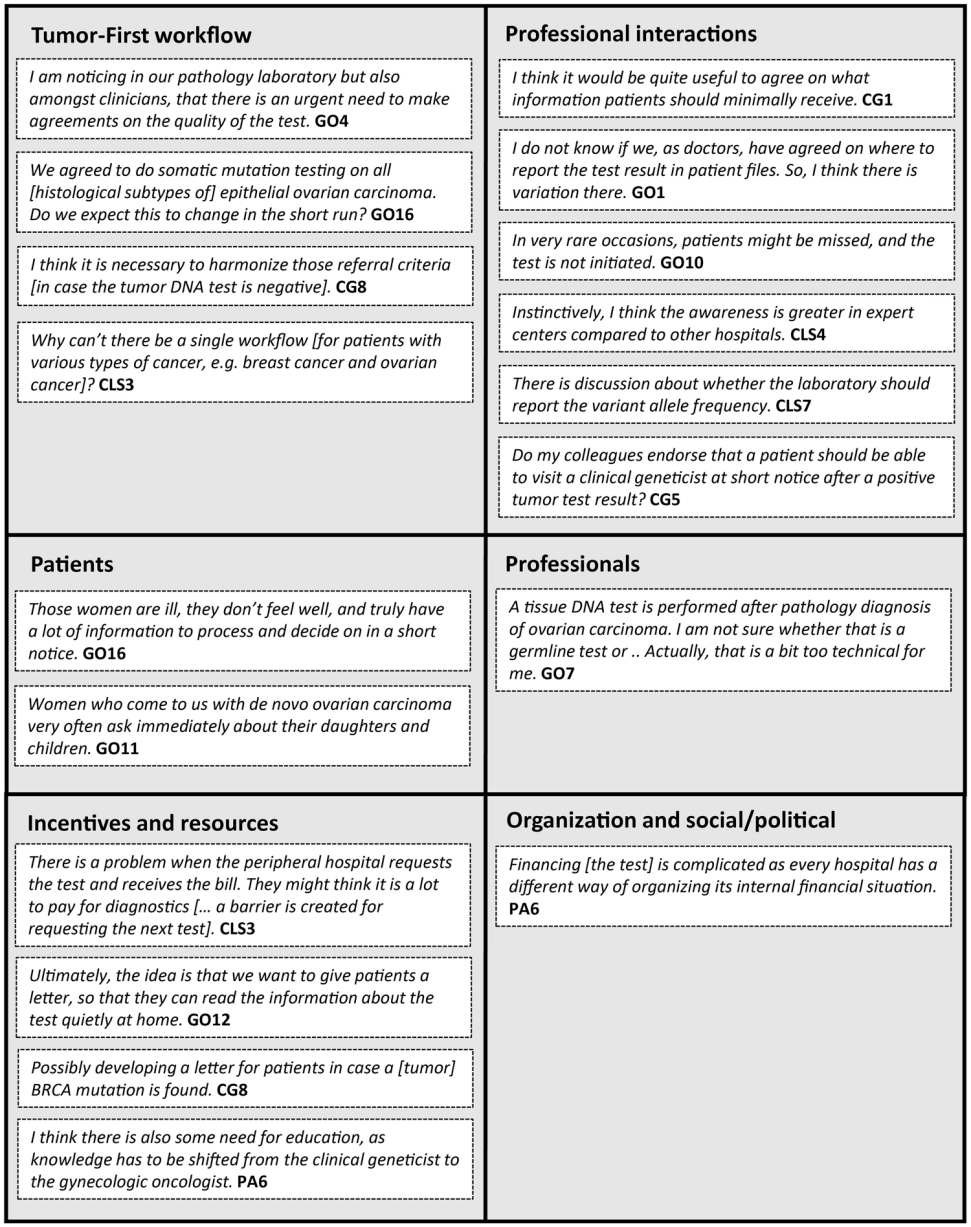
Examples of quotes mentioned by healthcare professionals during multidisciplinary focus group discussions. Quotes are organized based on the framework of Flottorp, at the level of the Tumor-First workflow, professional interactions, patients, professionals, incentives and resources, and organization and social/political. *GO* gynecologic oncologist, *PA* pathologist, *CG* clinical geneticist, *CLS* clinical laboratory specialist

The level of experience with the Tumor-First workflow varied among centers and professionals. The willingness and need to help those with less experience emerged from the focus group discussions. Furthermore, factors critical for implementation of the Tumor-First workflow were discussed, with a focus on how to promote implementation (what do healthcare professionals need?). Table 2 provides a summary of these themes, and Fig. 3 presents example quotes.

**Table 2:** Factors important in implementation of the Tumor-First workflow, and the needs of healthcare professionals

Domain	Theme	What do healthcare professionals need?
Innovation	Quality of the tumor DNA test	Advice and specification per laboratory
	Technical questions regarding the tumor DNA test (e.g., gene panel, histological triage, tissue material)	Advice and answer to questions
	Referral criteria in case of a normal tumor DNA test result (e.g., family history)	Advice and uniformity
	Responsibility initiating the tumor DNA test: gynecologist/ pathologist? Variation among centers	Advice
	Consistency with other workflows in cancer diagnostics (e.g., breast cancer)	Whenever possible alignment with other workflows
Professional interactions	Informing patients about Tumor-First process: What information? Informed consent?	Advice and minimal set of information (smart phrase), opt-out option
	Reporting test results in electronic medical records: clarity and findability on the long term	Uniformity and agreements
	Communication between laboratory and clinics: clarity of reporting	Advice and uniformity
	Communication of tumor DNA test result to medical oncologist, general practitioners, patients (and referral in case of aberrant result)	Center specific agreements on who is responsible, potentially fixed moments
	Time between aberrant tumor DNA test result and appointment at clinical geneticist	Clinical geneticist sees patient on short notice
	Avoid missing inclusion of patients during: initiation of test, referral to clinical geneticist	Building in checks, advice on how to monitor process
	Cooperation with non-expert hospitals: alertness initiation test and collaboration with different expert centers depending on the discipline	Assistance when implementing the workflow in non-expert centers
Patients	Variation in preferences/needs of patients: vulnerable and in middle of diagnostic process, but also questions regarding heredity	Align communication to individual patient needs
Professionals	Knowledge technical aspects tumor DNA testing Knowledge current center specific workflow	Training/ education Interdisciplinary communication within centers
Incentives and resources	Less incentive to initiate tumor DNA test if finances are a problem	National solution for financing test
	Assistance for clinicians: information material for patients, letter explaining tumor DNA test result, tool family history, training professionals	Assistance
	Assistance in communication to regional (non-expert) hospitals	Assistance
Organizational	Every hospital has its own internal procedures of division of finances	National solution for financing test
Social/political	Healthcare insurance arrangements	National solution for financing test

Factors are organized based on the framework of Flottorp

### Tumor-First workflow: need for advice and standardization

Besides the overall endorsement for implementation, healthcare professionals emphasized points for improvement related to the Tumor-First workflow. First of all, all professionals agreed that the quality of the tumor DNA test is of critical importance for the reliability of the workflow’s outcome. As the tumor DNA test is much more laborious and complex compared to the germline test, minimal criteria for the quality of the tumor DNA test are essential for all professionals to trust the results. Thus, it has to be clarified which laboratories are able to perform a tumor DNA test of sufficient quality. Completely aligning test quality among laboratories is complicated, however, due to variation in techniques. There is need for general parameters on the minimal quality of the test.

Additionally, with regards to the tumor DNA test, some technical questions were raised. Firstly, it was questioned which genes the tumor DNA test should cover in addition to *BRCA1* and *BRCA2,* since the germline test panel includes more OC risk genes. Secondly, a question arose regarding histological triage, specifically whether it is possible to select OC patients for tumor testing based on their histology. Thirdly, the tumor DNA test is performed on formalin-fixed paraffin-embedded tissue and fixed cytological material, but there was some ambiguity as to which tissue types are most suitable to be tested; e.g., resections versus biopsies and cytological material. Advice has to be prepared by experts in the field to answer these questions and provide clarity for all professionals involved.

Another theme that emerged entails referral criteria in case of a normal tumor DNA test result. Healthcare professionals indicated there could be additional reasons to refer a patient for genetic counseling, for example a family history of certain types of cancer or incomplete test results due to technical limitations. A concern was expressed that gynecologic oncologists might be less aware of a family history suggestive of familial cancer due to the new Tumor-First workflow. There is a need for an establishment of national referral criteria to the clinical geneticist in case of a normal tumor DNA test result, and healthcare professionals have to be aware of these criteria.

Lastly, there appeared to be variation in the professional discipline initiating the tumor DNA test in the different centers. Some centers are used to the pathologist routinely initiating the test after diagnosis of OC, whereas in other centers an active request by the gynecologic oncologist is necessary. It remains questionable whether this should be harmonized, as initiation in itself is more important than by whom the test is initiated. It would be beneficial to establish which professional discipline initiating the test best facilitates adherence to the workflow.

### Multidisciplinary professional interactions: exchange of information

At the level of professional interactions, healthcare professionals indicated it to be essential to keep everyone sufficiently informed on the different phases of the workflow and the results. This includes exchange of information among healthcare professionals from different disciplines, but also between healthcare professionals and patients.

Firstly, there appeared to be variation amongst gynecologic oncologists in what information they provide to patients regarding initiation of the tumor DNA test. Also, opinions on the amount of information a patient should receive varied, similar to opinions on whether or not explicit (written) consent is necessary for performing a tumor DNA test. In general, professionals agreed that patients must receive sufficient information on the tumor DNA test before they can consent to this test. To ensure this, there is a need for a minimal set of information that is standardly explained to a patient. Additionally, an opt-out option has to be incorporated into the workflow to provide the opportunity for patients to decide to not be included in a tumor DNA test procedure.

Another theme focuses on reporting the test result in electronic medical records, being another task of the gynecologic oncologist. Healthcare professionals agreed on the importance of clearly reporting the tumor DNA test result, and it has to be distinguishable from a germline test result. They also emphasized the importance of the findability and accessibility of the tumor DNA test result in the long term, indicating that current electronic medical records generally fail in this regard. There appeared to be a need for agreement on where and how to report the tumor DNA test result in commonly used electronic health record systems (e.g., HiX and Epic).

Additionally, communication between the clinical laboratory specialists and gynecologic oncologists emerged as a relevant theme. The manner in which clinical laboratory specialists and pathologists report the tumor DNA test result can help clinicians interpret the clinical consequences of the result and can avoid misinterpretations. For example, it was indicated that it might cause misinterpretations if a clinical laboratory specialist reports the presence of *TP53* pathogenic variants, as these should be considered as somatic in this context and do not require any action on the part of the gynecologic oncologist. There is a need for uniform guidance on how clinical laboratory specialists should report the tumor DNA test result to clinicians.

Furthermore, the responsibility of informing patients about the tumor DNA test result has to receive special attention, similar to the responsibility of communication to medical oncologists and general practitioners. There appeared to be variation among centers in who takes this responsibility (mostly the gynecologic oncologist). Center-specific and region-specific agreements are essential in this regard. Moreover, clinical geneticists have to be able to see patients with an aberrant tumor DNA test on short notice, to avoid any unnecessarily long period of uncertainty for the patient.

### Multidisciplinary professional interactions: monitoring patient inclusion

Monitoring patient inclusion in the Tumor-First workflow was an important topic discussed related to the level of professional interaction. Healthcare professionals emphasized the importance of including all patients in the Tumor-First workflow. Patients should not be missed, neither during the initiation of the tumor DNA test nor the referral for germline testing (based on an aberrant tumor DNA test result). Designing checks during several points of the workflow was proposed as a safety guard. Monitoring patient inclusion in the Tumor-First workflow may be essential both at a local and national level.

Additionally, the level of awareness in non-expert centers has to receive specific attention. In comparison to gynecologic oncology centers, there seems to be less awareness in non-expert centers about initiating a tumor DNA test. Also, professional collaboration among disciplines is more complicated in non-expert hospitals, as they sometimes collaborate with multiple expert centers, i.e. they collaborate with one center for a gynecologic oncology expert and with another for a clinical genetics expert.

### Implementation factors at the level of patients and professionals

At the level of patients, healthcare professionals perceive variation in the needs and preferences of patients regarding the Tumor-First diagnostic workflow. Considering that patients are ill, vulnerable and in the middle of the diagnostic process, one must avoid information overload and not provide them with too much information on the tumor DNA test. On the other hand, some patients come up with questions regarding heredity and cancer risk for family members, in which case healthcare professionals should always consider the needs and preferences of patients on an individual basis when providing them information.

At the level of professionals, it stood out that a small number of healthcare professionals have insufficient knowledge on the technical aspects of the tumor DNA test; particularly the terms ‘somatic’ and ‘germline’ caused confusion. Educational training would be helpful in this regard. Moreover, some healthcare professionals were unaware of the specific activities of other professionals within the current workflow for OC patients. Interdisciplinary communication within centers can be encouraged.

### Incentives and resources, organizational support and national healthcare policy

When focusing on incentives and resources, healthcare professionals mentioned that hospitals (specifically non-expert centers) have less incentive to initiate a tumor DNA test if they need to pay for the test themselves. Therefore, it is of critical importance that a national solution for financing of the tumor DNA be established. This national solution is complicated by healthcare insurance arrangements (social/legal level) as well as by the fact that every hospital has its own internal procedures of division of finances (organizational level).

Additionally, at the level of incentives and resources, clinicians (predominantly gynecologic oncologists) would appreciate assistance during the implementation of the workflow. Information material for patients, letters explaining the tumor DNA test results and a tool for assessing family history were examples of assisting material that would be useful. Also, educational training for professionals and assistance when communicating to non-expert centers would be of added value.

## Discussion

Multidisciplinary focus group discussions have revealed several factors important to promote the implementation of the Tumor-First workflow among OC patients. Gynecologic oncologists, pathologists, clinical geneticists, and clinical laboratory specialists endorse large-scale implementation of this new workflow. Tumor-First is generally accepted as a beneficial workflow because it efficiently provides information both on heredity for patients and their family members and on treatment response, and also because it facilitates communication of genetic risks quite soon after diagnosis. However, some factors need specific attention during implementation of the workflow. Firstly, specific standardization and advice on the logistics of the Tumor-First workflow proved necessary. Additionally, keeping each healthcare professional sufficiently informed on the process and results of the Tumor-First workflow was seen as a challenge. Thirdly, healthcare professionals agreed on the importance of monitoring the inclusion of all OC patients. Overall, healthcare professionals would appreciate assistance in implementation and monitoring the outcome of the Tumor-First workflow in the daily work routine.

Internationally, research on tumor DNA testing for OC patients is ongoing [21–23] and different countries prefer varying workflows, including parallel testing [24]. In the Netherlands, this variation was similarly present, where regions differed in their preferred workflows [3, 25]. Currently, the Tumor-First workflow is preferred and differences will be aligned. Two validation studies have shown a tumor DNA test being able to reliably detect germline pathogenic variants [26, 27], but it remains important to build up trust in the quality of the tumor DNA test. Also, more detailed standardization and agreements regarding the workflow proved necessary, for example which minimal set of genes to test for in addition to *BRCA1* and *BRCA2.* The tumor DNA test has to be expanded to include other OC risk genes, as healthcare professionals and other studies (e.g., [23]) have expressed concerns about solely testing for pathogenic variants in *BRCA1* and *BRCA2*. National agreements and guidelines should be established, and the balance between reducing and allowing variation between centers has to be carefully considered. Ultimately, finances could make or break successful implementation of the Tumor-First workflow. As some centers experience issues in financing the tumor DNA test, it is of utmost importance that a national solution be established.

Factors important for implementation of the Tumor-First workflow take place in a wider context of current shifts in (i) the centralization of specialized oncology care in the Netherlands [19] and (ii) the relation between clinical genetics and multidisciplinary oncology teams. In recent years, oncologists and other medical specialists are increasingly ordering tumor DNA tests or germline tests without directly consulting a clinical geneticist, which has been dubbed ‘mainstreaming’ genomic and genetic testing [28–30]. Complicatedly, these new approaches touch upon issues of informed consent, which were also discussed extensively during our focus group interviews. Healthcare professionals agreed that while written consent remains necessary for germline testing, oral consent is adequate for Tumor-First testing, considering that (i) a minimal set of information has to be communicated to patients; (ii) individual patient needs have to be taken into account; and (iii) patients must have the possibility to opt-out. This approach of verbal discussion has been similarly proposed by others [24, 31] and resembles the approach of ‘layered consent’, recently introduced in a perspective on informed consent for mainstreaming genomic sequencing [28].

A strong aspect of our study was the high rate of participation in focus groups amongst healthcare professionals from all disciplines (around 75%). The lower threshold for participation was a benefit of the online design of our study, however, performing focus groups online might have hindered the interaction during discussions. Implementation factors were identified from a broad perspective, but our limited sample size and center-specific variation within the Netherlands did not allow for subgroup analyses. OC patients were not present during this study, as patient experiences have been previously obtained during a regional pilot study [3]. In consultation with patient organizations, it was decided to primarily focus on implementation factors from a healthcare professional perspective here and to involve patients during the development of the implementation tools (e.g., patient information explaining the process).

The use of our findings can be twofold. Firstly, the findings of the focus group interviews will be used in developing a tailored implementation strategy for the Tumor-First workflow. An implementation toolbox will be developed based on the factors identified during the focus group interviews. This toolbox shall contain information to support healthcare professionals to change their practices and work methods according to the Tumor-First workflow, for example: advice and consensus documents on specific topics, suggested actions for optimization of the workflow, and supporting materials for clinicians (e.g., letters explaining the procedure to hand out to patients). Additional expert meetings will be necessary for the development of the content of this toolbox. Previous research has shown that an implementation toolbox effectively broadened the mindset of healthcare professionals on how to improve their practice(s) [32]. Future research will elucidate the effectivity of the toolbox in the implementation of the Tumor-First workflow.

Secondly, implementation factors and methods may be useful for the implementation of future workflows where multiple professionals collaborate. The Tumor-First workflow for OC patients is the second example of using tumor testing as a prescreen for heredity testing. The first being universal microsatellite instability testing of colorectal tumors as a prescreen for hereditary colorectal cancer, i.e. Lynch syndrome. Previous research on this topic, interview-based and survey-based, highlighted an interdisciplinary approach during implementation as an important factor [33, 34]. Here, we have discussed implementation factors from a multidisciplinary perspective, as this is in line with collaboration necessary for effective implementation. In the future, it is to be expected that the practices of tumor DNA testing (as a prescreen for heredity) will be expanded to include various other cancer types, for example prostate and pancreatic cancer [35–37]. The challenges identified during our focus group discussions, such as keeping all healthcare professionals informed and monitoring the process of including all eligible patients, will also apply there.

In conclusion, this study using multidisciplinary focus group interviews has revealed that healthcare professionals endorse implementation of the Tumor-First workflow in general, but some aspects require specific attention. Using these results to develop a tailored implementation strategy will facilitate the Tumor-First workflow to optimize integrated care for epithelial ovarian cancer patients. Additionally, our results and research strategy may be useful for the implementation of future innovations influencing the interdisciplinary collaboration of clinical geneticists with oncology teams.

## Data Availability

The data (Dutch language) is available upon reasonable request to the authors.
